# Biomechanical evaluation of a novel dualplate fixation method for proximal humeral fractures without medial support

**DOI:** 10.1186/s13018-017-0573-4

**Published:** 2017-05-12

**Authors:** Yu He, Yaoshen Zhang, Yan Wang, Dongsheng Zhou, Fu Wang

**Affiliations:** 1 Department of Orthopaedics, Peking Union Medical College Hospital, Chinese Academy of Medical Sciences and Peking Union Medical College, No. 1 Shuaifuyuan Wangfujing, Beijing, 100010 China; 2 Department of Orthopedics, Beijing Chaoyang Hospital, Capital Medical University, No. 8 Gongti Nanlu, Beijing, 100020 China; 3 Department of Medical Laboratory Diagnosis Center, Jinan Central Hospital, No. 105 Jiefang Road, Ji’nan, 250014 Shandong China; 4 Department of Orthopedic Surgery, Shandong Provincial Hospital Affiliated to Shandong University, No. 324 Jingwu Road, Ji’nan, 250021 Shandong China

**Keywords:** Proximal humeral fractures, Medial support plate, Varus malunion, Biomechanical characteristics, Finite element analysis

## Abstract

**Background:**

Comminuted fractures of the proximal humerus are generally treated with the locking plate system, and clinical results are satisfactory. However, unstable support of the medial column results in varus malunion and screw perforation. We designed a novel medial anatomical locking plate (MLP) to directly support the medial column. Theoretically, the combined application of locking plate and MLP (LPMP) would directly provide strong dual-column stability. We hypothesized that the LPMP could provide greater construct stability than the locking plate alone (LP), locking plate combined with a fibular graft (LPSG), and locking plate combined with a distal radius plate (LPDP).

**Methods:**

LP, LPMP, LPSG, and LPDP implants were instrumented into the finite element model of a proximal humeral fracture. Axial, shear, and rotational loads were applied to the models under normal and osteoporotic bone conditions. The whole simulation was repeated five times for each fixator. To assess the biomechanical characteristics, the construct stiffness, fracture micromotion, stress distribution, and neck-shaft angle (NSA) were compared.

**Results:**

The LPMP group showed significantly greater integral and regional construct stiffness, and endured less von Mises stresses, than the other three fixation methods. The stresses on the lateral locking plate were dispersed by the MLP. The LPMP group showed the least change in NSA.

**Conclusions:**

From the finite element viewpoint, the LPMP method provided both lateral and medial direct support. The LPMP system was effective in treating proximal humeral fracture with an unstable medial column.

## Background

Comminuted and displaced fractures of the proximal humerus are common injuries, which are typically encountered in elderly patients with osteoporosis [1, 2]. There are numerous fixation methods available [3–6], including the locking plate (LP) system and intramedullary nailing. The LP system is widely preferred in treatment of these fractures, and clinical results are satisfactory [7–9]; however, the complications of varus malunion and screw perforation still occur in up to 16.3 and 7.5% of cases [10], respectively, even when calcar screws are used.

Failure of medial support can lead to varus deformity, followed by screw penetration, plate breakage, impingement, restricted motion, and early arthrosis [11–13]. Accordingly, the stability of the medial column is the key factor for successful treatment. Various methods based on the LP have been created to buttress the medial column. Gardner et al. [14] used a locking plate combined with an intramedullary fibular graft (LPSG) to provide additional medial support and prevent varus malunion; this technique was tested successfully in cases of proximal humeral fracture with unstable medial support [13–15]. Choi et al. [16] developed a dual-plate fixation technique for comminuted proximal humeral fracture in which a locking plate and the Variable Angle Locking Compression Plate Distal Radius System (LPDP) were used to prevent nonunion and varus collapse of the neck-shaft portion, which are caused by severe comminution.

**Fig. 1:**
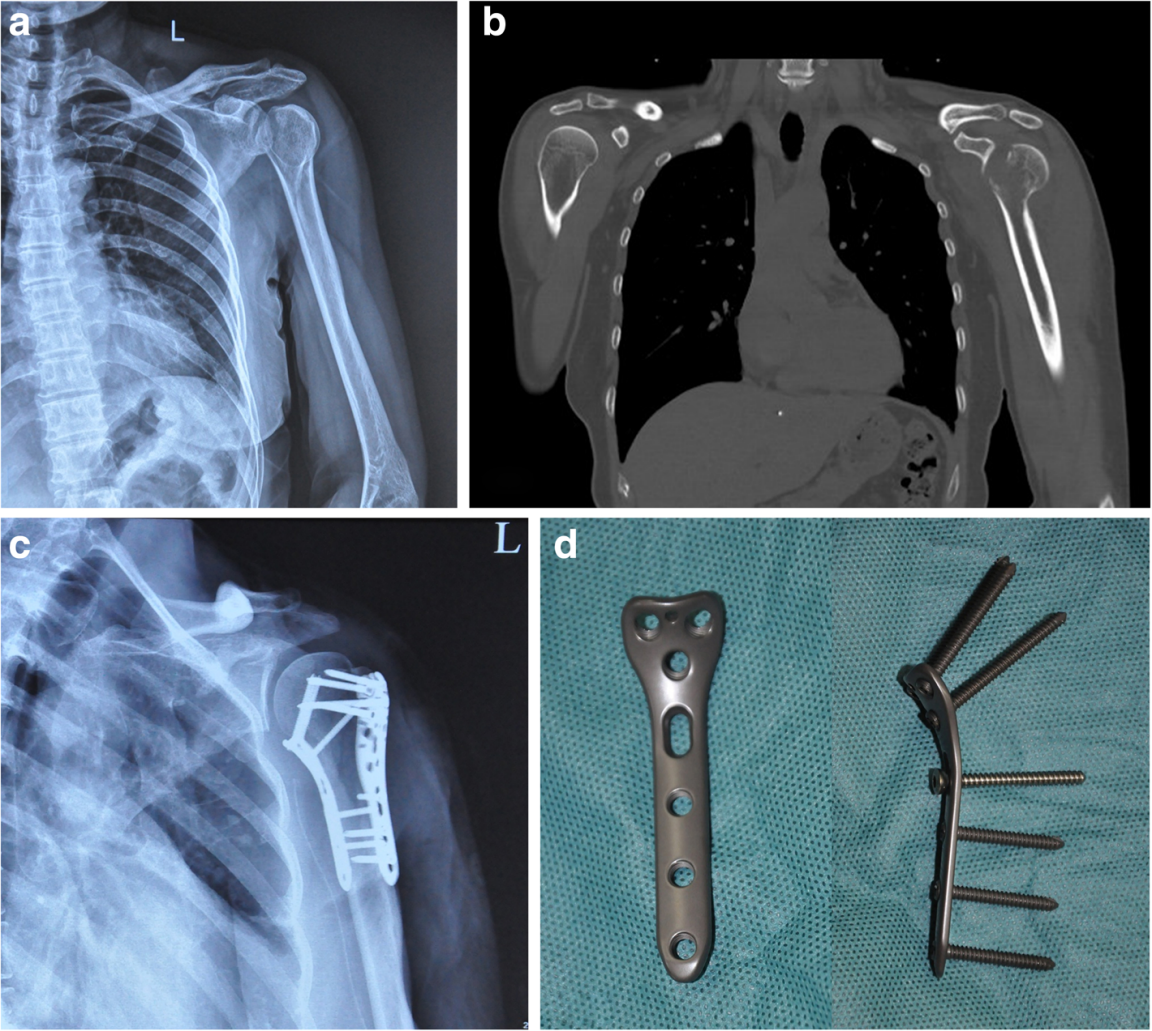
A 45-year-old female patient sustained a proximal humeral fracture with posterior dislocation of the shoulder joint. Preoperative anteroposterior radiographs (**a**) and computed tomography (**b**) scanning were performed. The medial column of the humerus was unstable (**b**). The postoperative anteroposterior radiograph (**c**) showed the application of the LPMP method (**d**)

From a biomechanical viewpoint, the LPSG provides direct medial support but not direct fixation, while the LPDP provides direct lateral fixation and indirect medial buttressing. We considered that direct medial support and fixation may be more effective in providing dual-column support and antirotational stability. Hence, we designed a novel medial anatomical locking plate (MLP) to directly support the medial column (Fig. 1d). Theoretically, the combined application of the MLP and a lateral locking plate (LPMP) would provide strong dual-column stability directly. Clinically, we have successfully performed LPMP fixation in cases of proximal humeral fracture with an unstable medial column (Fig. 1). However, the biomechanical performance of the LPMP fixation technique has not been investigated systematically.

We have to admit that this new generation of plate will cause extensive controversy. Our previous studies have proven that this method is clinically applicable. However, compared with other methods, the biomechanical property is not clear. In the current paper, we only discuss biomechanical properties from the view of finite element analysis. Therefore, the purpose of this study was to conduct finite element analysis of the biomechanical properties of four kinds of fixation (LP, LPMP, LPSG, and LPDP) in proximal humeral fracture with an unstable medial column.

## Methods

This study was approved by the Ethics Committee of our hospital. All patients provided written informed consent before study commencement.

### Finite element models and implants

**Fig. 2:**
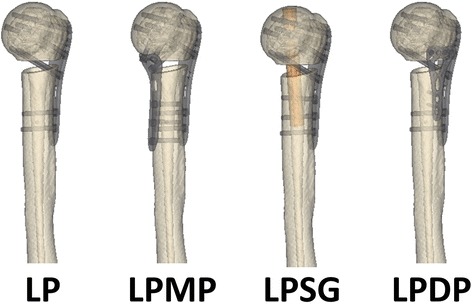
The proximal humeral fracture model with an unstable medial column was simulated as ladder-shaped bone defects below the surgical neck from the intact model. Four types of fixation configurations, LP, LPMP, LPSG, and LPDP, were positioned into the proximal humeral fracture model according to standard surgical guidelines

A three-dimensional finite element model was developed from a computed tomography scan (Lightspeed VCT, GE, Fairfield, CT) of a 38-year-old healthy female. This intact model was used to simulate a model of proximal humeral fracture with an unstable medial column as a ladder-shaped osteotomy below the surgical neck (Fig. 2). The medial and lateral bone defects were 10 and 5 mm, respectively. The bone stock was simulated in two conditions: normal bone (Nor) and osteoporotic bone (Ost). The elastic modulus of the Ost bone was decreased by 33% for cortical bone and 66% for cancellous bone. Four types of fixation configurations (LP, LPMP, LPSG, and LPDP) were positioned into the proximal humeral fracture model according to standard surgical guidelines [8, 14, 16–19] (Fig. 2). The LP (Waston Medical, China), the MLP (Waston Medical, China), and the Variable Angle Locking Compression Plate Distal Radius System (VA-LCP, Synthes, Switzerland) were respectively 90, 70, and 54 mm in length and 20, 18, and 22 mm in width. A hollow cylinder was used to simulate the fibular graft. The length, outer radius, and inner radius of the hollow cylinder were 85, 5, and 2 mm, respectively. Calcar screws were not used on the locking plate in the LPMP group due to this being a minimally invasive technique. The threads of the locking screws and cortical screws were omitted to simplify the models.

### Finite element analysis

The finite element analysis was performed in Abaqus 6.13 (3DS, Waltham, MA). Linear elastic isotropic material properties were assigned to all models and implant materials. The cortical bone and cancellous bone were assumed with Poisson’s ratio of 0.3 and elastic modulus of 13,800 and 1380 MPa, respectively [20]. The contact behavior of the plate/locking screw and locking screw/bone interfaces was defined as fully fixed. The cortical screws were fixed into the plates and cortices. The graft was fixed using four screws, including two proximal locking screws, one distal cortical screw, and one distal locking screw. The friction coefficients of implant/bone and graft/bone were 0.08 and 0.3, respectively. All of the contact elements were defined as deformable elements.

**Fig. 3:**
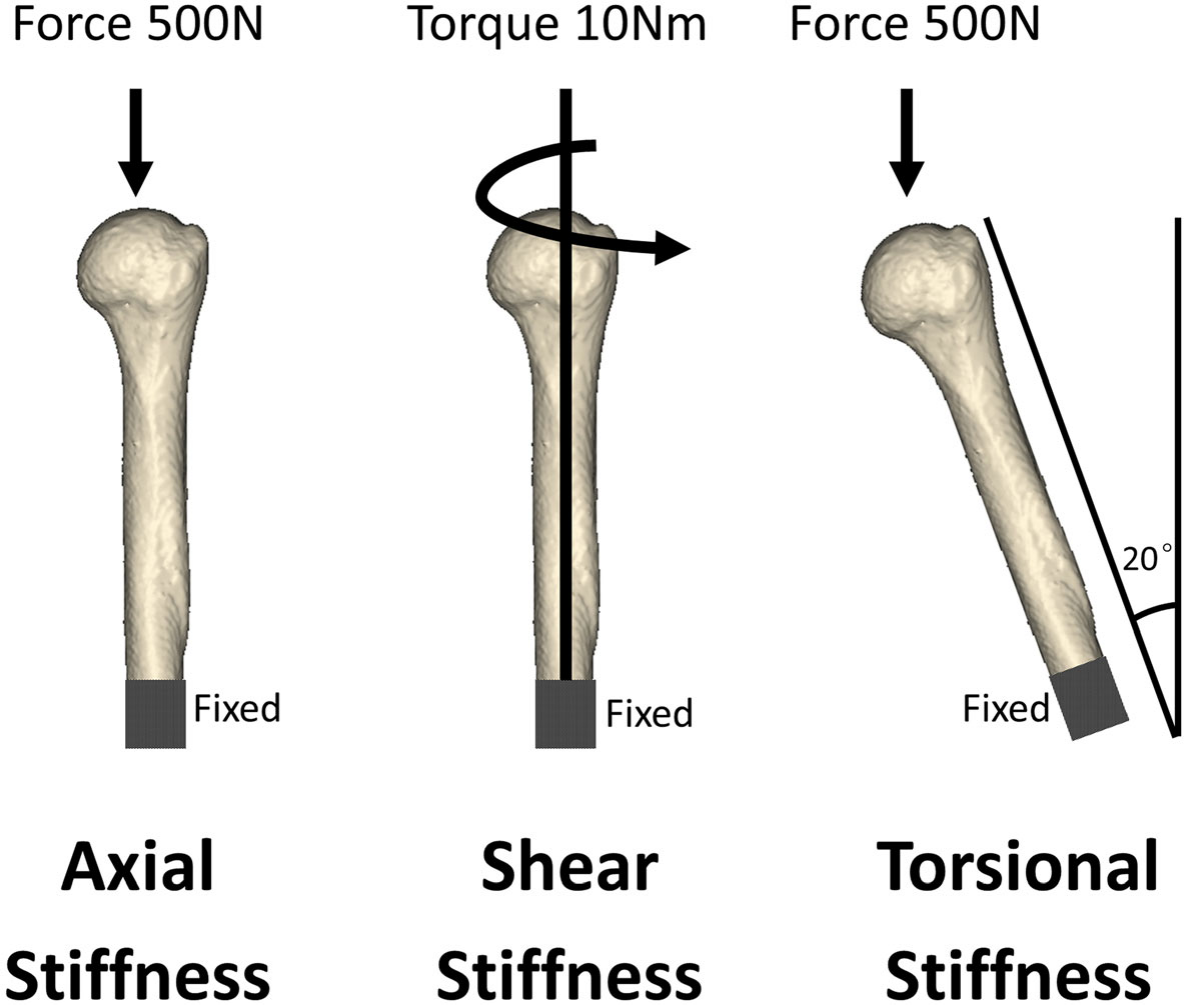
The distal segment of the humeral shaft was fixed. Axial force, shear force, and torsion were applied to the models

The distal segment of the humeral shaft was fixed (Fig. 3). Axial force, shear force, and torsion were applied to the models (Fig. 3). For axial force, 500-N loads oriented vertically in the coronal and sagittal planes were distributed onto the proximal humeral head. On the basis of axial conditions, the angle of the model was changed by 20° to simulate shear force. The shear force simulated the force that a proximal fracture site would experience while the patient was rising out of a chair or crutch weight-bearing. To simulate rotation, a 10-Nm torque was applied to the proximal humeral head around the axis of the humeral shaft. The whole experiment was repeated five times.

**Fig. 4:**
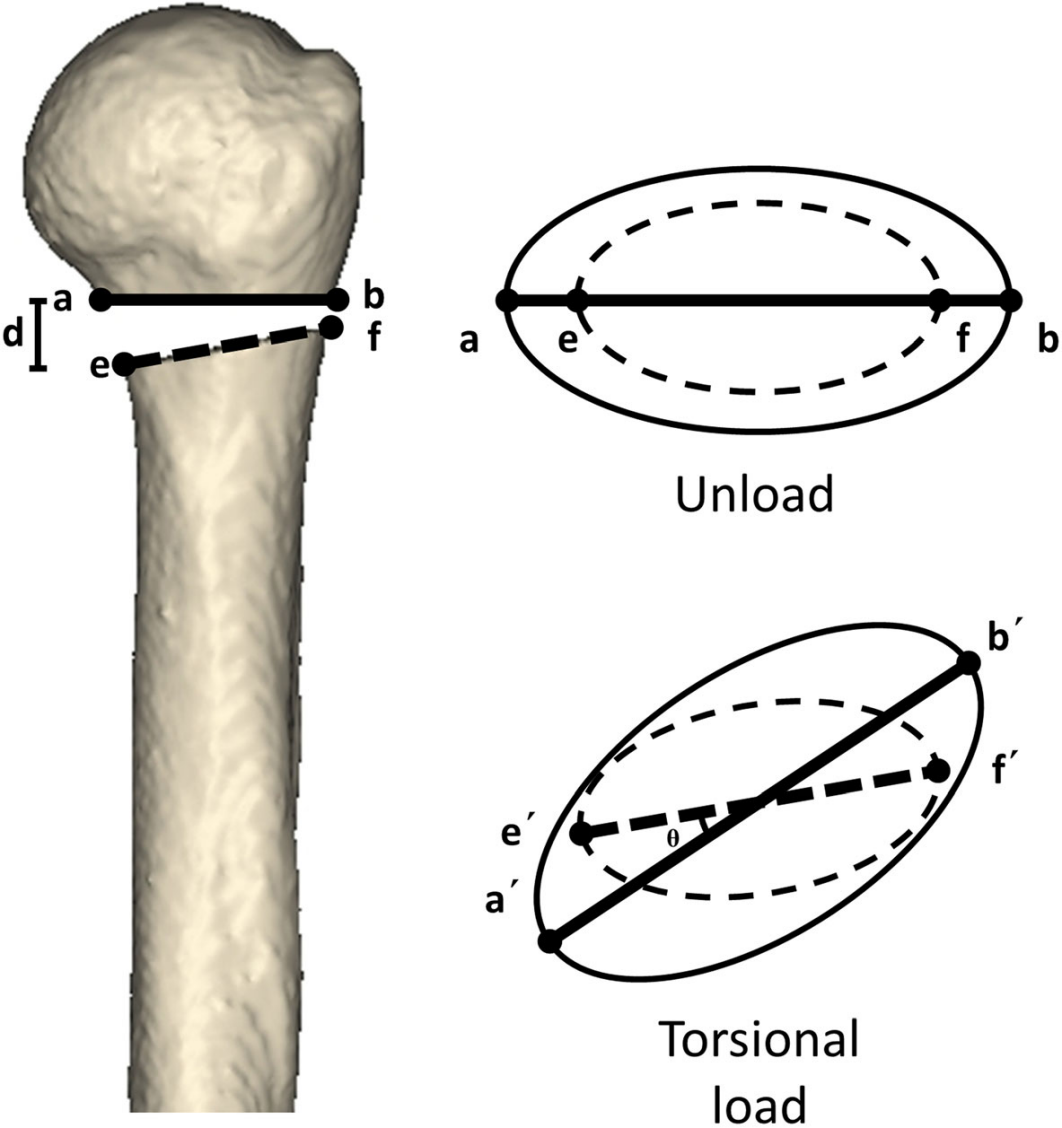
The stability of the fracture region under axial and shear loads was assessed as the variation of the distance of the medial fracture gap (*line d*). The angular variation between the proximal and distal fracture gap was determined to assess the regional rotational stability (*angle θ*)

This study aimed to compare the integral stability, fracture regional stability, and stress performance of four fixation methods within the immediate postoperative period. The construct stiffness was determined to compare the integral stabilities of the various bone-implant constructs. The stability of the fracture region under axial and shear loads was assessed as the variation in the medial fracture gap distance (Fig. 4). The angular variation between the proximal and distal fracture gap was determined to assess the regional rotational stability (Fig. 4). The neck-shaft angle (NSA) was assessed to evaluate the severity of the varus deformity. To assess the force conditions, the von Mises stress distribution and maximum stresses on the implants were determined.

### Statistical analysis

Statistical analyses were performed with SPSS (version 19.0; SPSS Inc, Chicago, IL) software. A Kolmogorov-Smirnov test was used to analyze normal distribution, and the Bartlett test was used to analyze homogeneity. A separate one-way analysis of variance (ANOVA) was performed. Comparison of the maximum von Mises stress of implants between the MLP and the VA-LCP methods was obtained using an independent-sample *t* test. The level of statistical significance was defined as *p* < 0.05.

## Results

### The construct stiffness

**Fig. 5:**
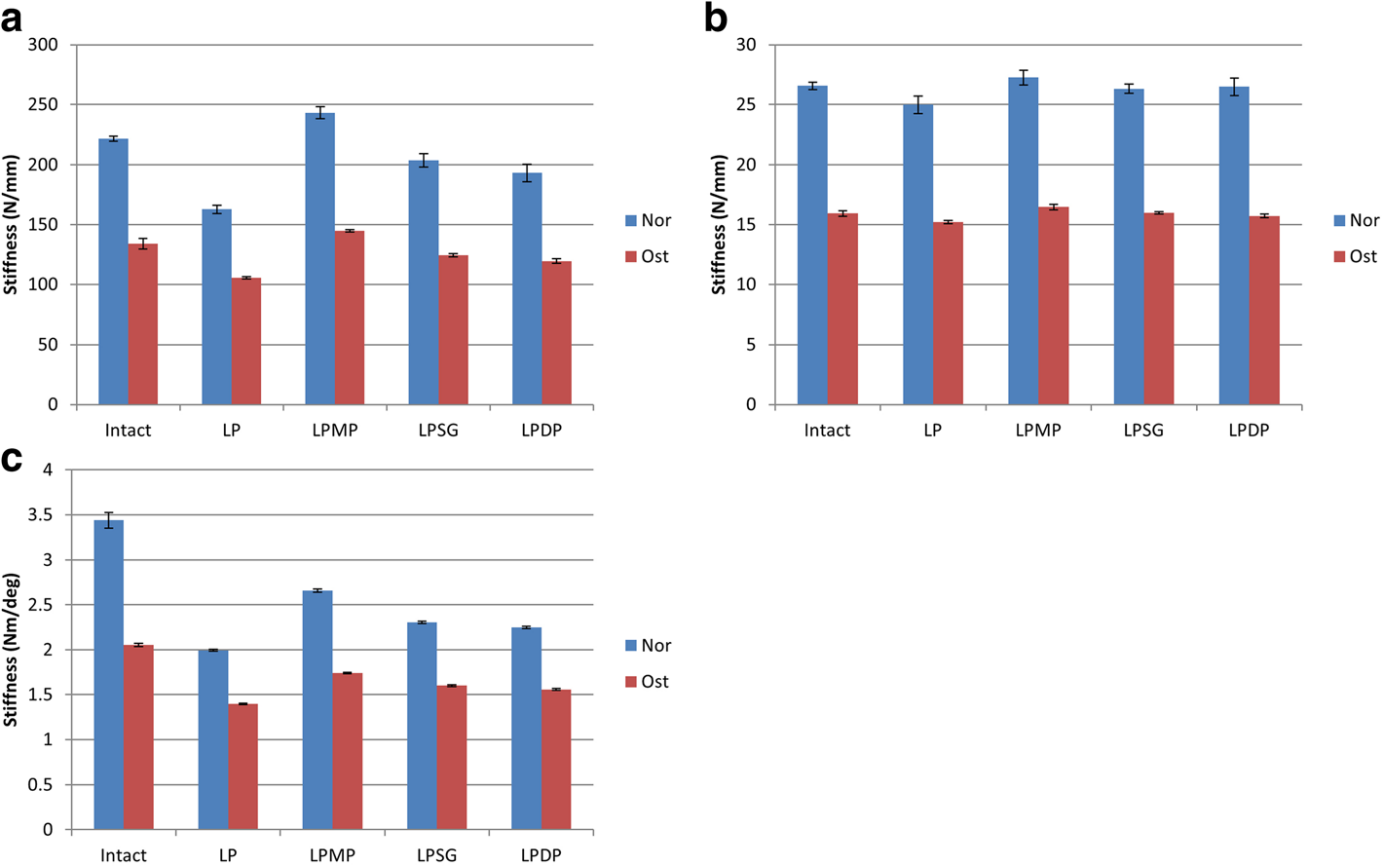
The results of the construct stiffness within axial (**a**), shear (**b**), and torsional (**c**) loads are shown

The results of the construct stiffness were shown in Fig. 5. In general, the construct stiffness was markedly decreased when bone stock was reduced. For axial loads, the LPMP group showed significantly greater construct stiffness compared with the LP, LPSG, and LPDP groups under both Nor and Ost conditions (*p* < 0.03). The axial stiffness of the reconstructed models in the LPMP group was actually 109.8% of the intact model under Nor conditions and 108.0% of the intact model under Ost conditions. Under Nor conditions, the shear stiffness did not significantly differ between groups (*p* > 0.19), except that the shear stiffness of the LP group was significantly lesser than that of the three other groups (*p* < 0.02). The shear stiffness of the construct was slightly greater in the LPMP group than in the LP, LPSG, and LPDP groups; however, this difference was not statistically significant. For torsional stiffness, all comparisons between groups showed statistical differences under both bone conditions (*p* < 0.01), except when comparing LPSG with LPDP under Nor conditions (*p* = 0.45).

### Fracture regional stability

**Fig. 6:**
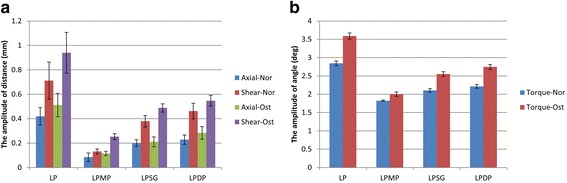
The results of the amplitude of distance (**a**) and angle (**b**) are displayed

Figure 6 showed the results of the amplitude of distance and angle. Under Nor conditions, the LPMP group showed significantly less micromotion than all the other groups (*p* < 0.007). The amplitudes of the medial fracture gap distances under axial and shear loads were 4.9 and 5.4 times less in the LPMP group than in the LP group. No significant differences were found between the LPSG group and the LPDP group (*p* > 0.877). Under Ost conditions, the changes in fragment gap distance under the axial and shear load conditions were not significantly different between the LPMP, LPSG, and LPDP groups (*p* > 0.119). For the angular variation between the proximal and distal fracture gap, all comparisons between groups showed significant differences under both bone conditions (*p* < 0.01). The LPMP method provided superior antirotational stability compared with all the other methods.

### Neck-shaft angle

**Fig. 7:**
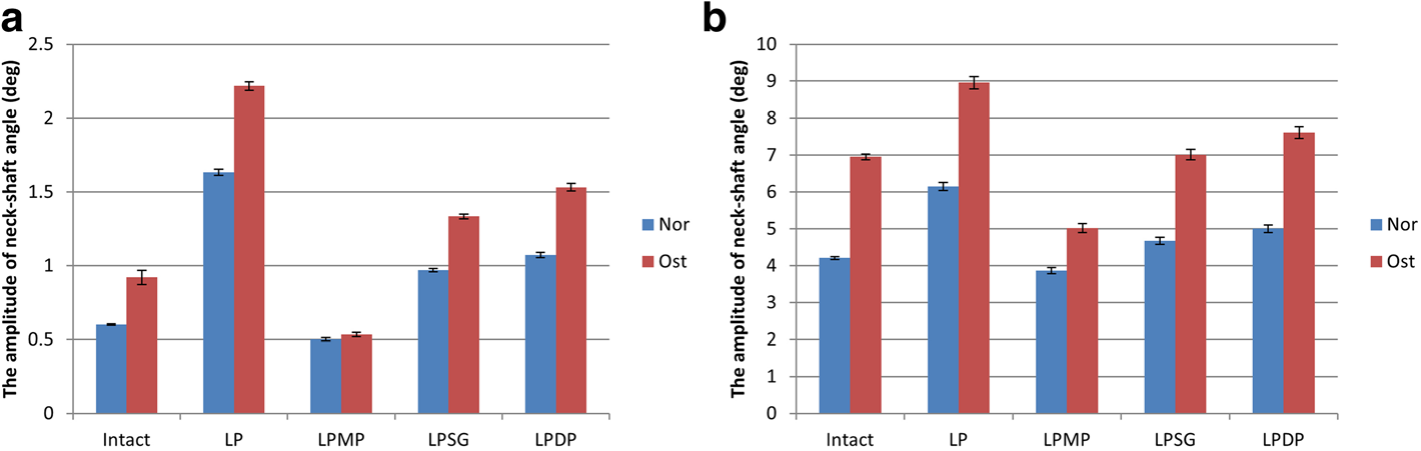
The results of the amplitude of NSA under axial (**a**) and shear (**b**) load conditions are shown

The results of the amplitude of NSA are shown in Fig. 7. Under the axial and shear loads, the amplitude of the NSA under Nor conditions was significantly different between all groups (*p* < 0.01). The LPMP group showed significantly less change in NSA than all the other groups. Similar results were obtained under Ost conditions, except when comparing the shear load of the intact group with the LPSG group (*p* > 0.05).

### The von Mises stress

**Fig. 8:**
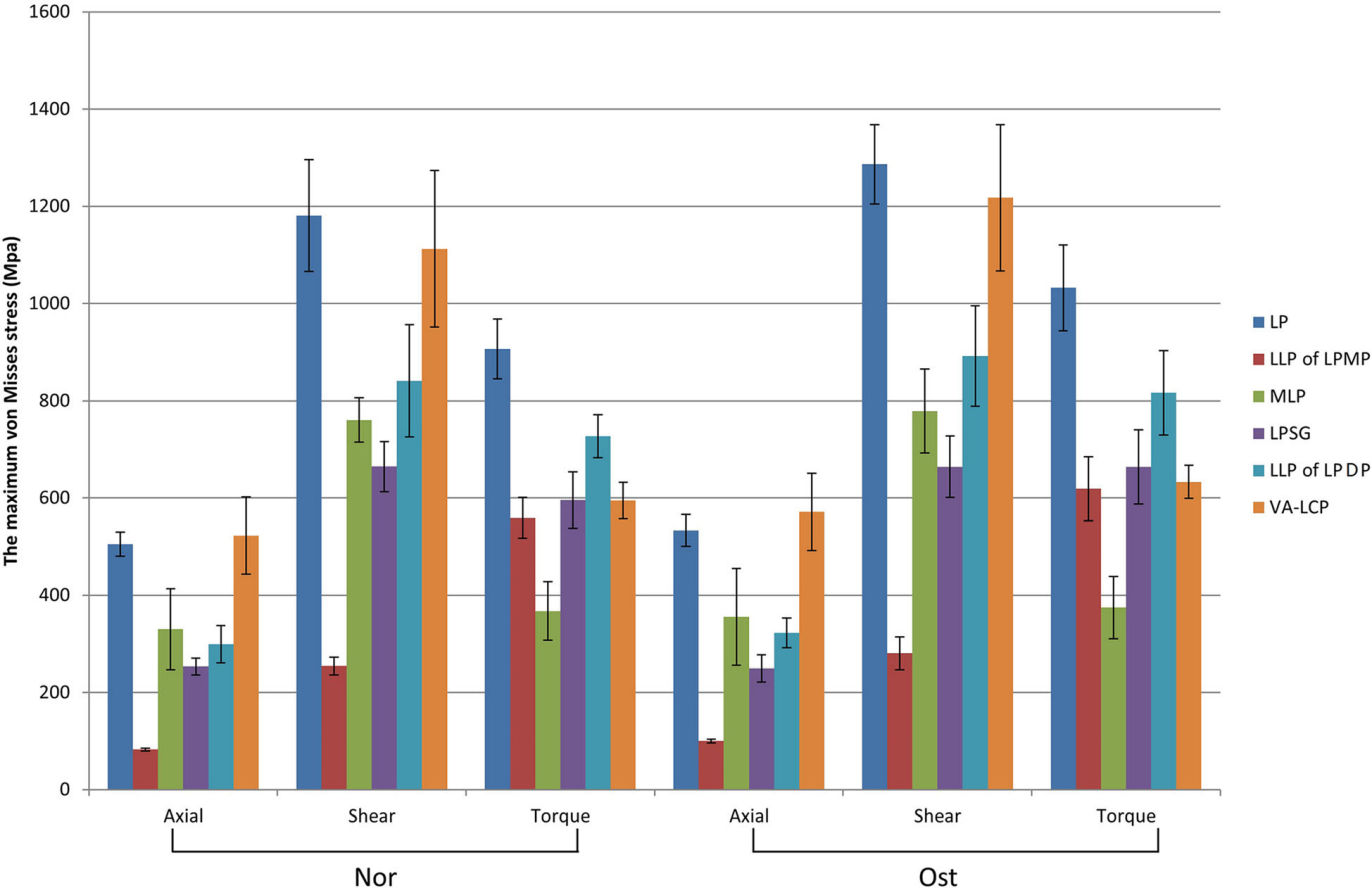
The maximum von Mises stresses of the LP, LPMP, LPSG, and LPDP are shown

**Fig. 9:**
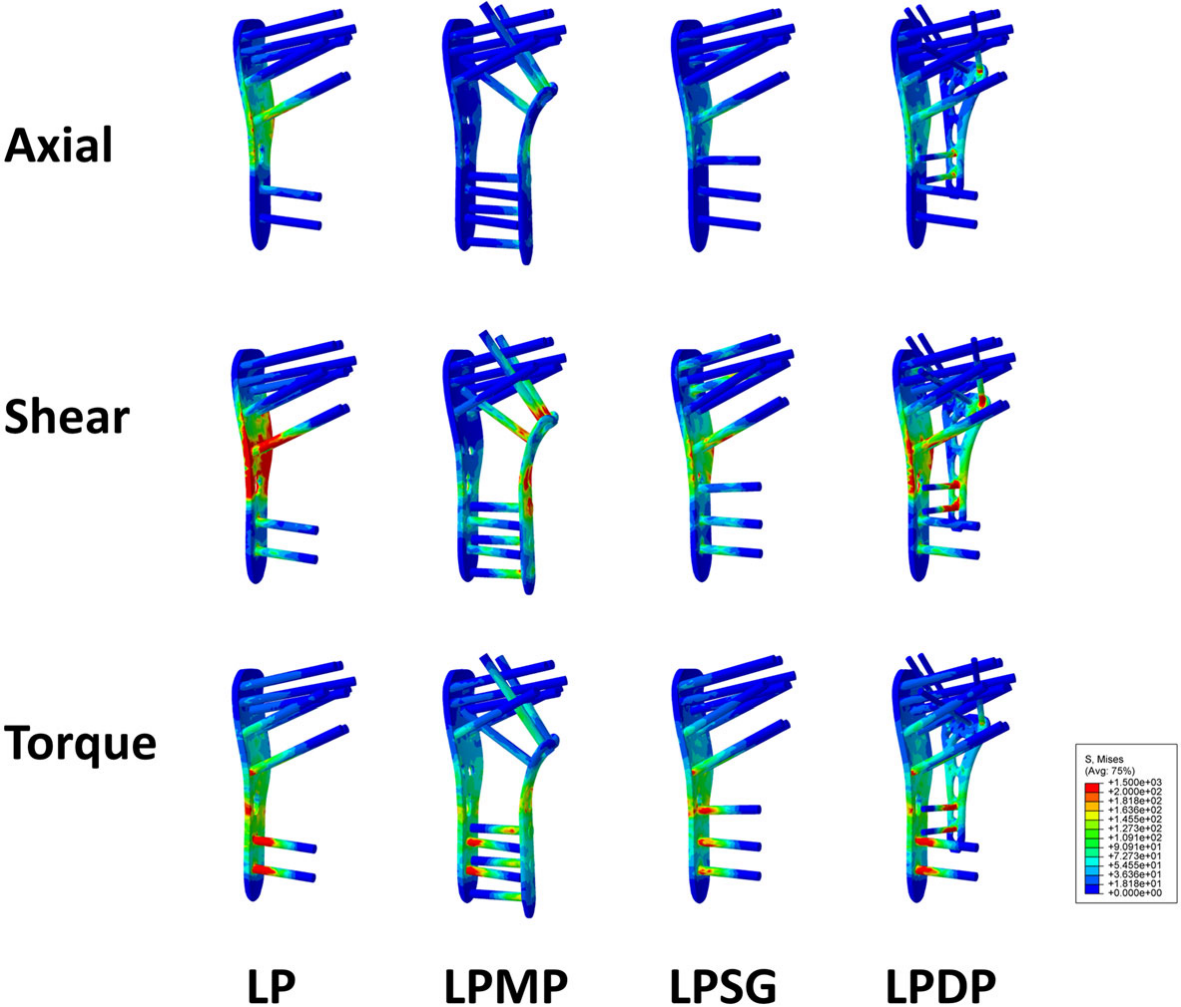
The maximum von Mises stress distributions of the LP, LPMP, LPSG, and LPDP within normal bone condition are shown

The maximum von Mises stress and the stress distribution of the implants are shown in Figs. 8 and 9, respectively. The greatest von Mises stress of the implants under all load types occurred in the LP group, while the LPMP group experienced the least von Mises stresses. All comparisons between groups showed significant differences (*p* < 0.01), except for the comparison of the axial loads of the LPSG group versus the LPDP group under Nor conditions (*p* = 0.055) and the torsional load of the LPMP group versus the LPSG group under both bone conditions (*p* > 0.05).

**Fig. 10:**
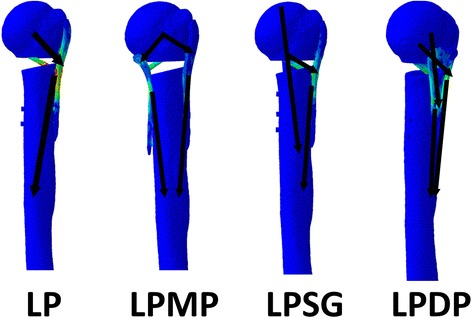
The mechanical pathways of the constructs under axial load condition are shown

In the LP group, the stress was concentrated around the fracture site, especially on the calcar screws and plate-calcar screw joints (Fig. 9). In the LPMP, LPSG, and LPDP groups, the stresses were dispersed largely by additional implant/graft from locking plates. Consequently, the MLP and VA-LCP endured more stress, but the graft survived. The von Mises stress distribution indicated that the mechanical pathway of the LPMP and LPSG methods was dual-column conduction, and non-center symmetrical conduction in the LP and LPDP methods (Fig. 10). The von Mises stress was similar under both Nor and Ost conditions.

## Discussion

Internal fixation of unstable displaced proximal humeral fracture remains difficult, especially in patients with osteoporosis. This type of fracture has been successfully treated by the proximal humeral lateral LP [7–9]; however, the associated rate of complications is 49%, with varus malunion being the most common complication [10]. Failure of medial support can lead to varus deformity, followed by screw penetration, plate breakage, impingement, restricted motion, and early arthrosis [11–13]. Medial column stability is essential for successful outcome. Various methods based on the LP have been created to support the medial column. The LPSG reportedly provides additional medial support and prevents varus malunion [14]. The advantages of the LPSG have been proven clinically and biomechanically in proximal humeral fracture with unstable medial support. However, the drawbacks of LPSG include technical difficulty, limited supply, infection risk, and disease transmission risk. Dual-plate fixation has been used for comminuted proximal humeral fractures [16]. The LPDP system is used to prevent malunion and varus collapse of the neck-shaft portion, which are caused by severe comminution; however, only a limited number of patients have undergone this operation, and the biomechanical properties have not been investigated.

The LPSG provides direct dual-column support and partial torsional stability without providing direct medial fixation, while the LPDP provides direct lateral fixation and indirect medial buttressing. Direct medial support and fixation may provide more effective dual-column support and antirotational stability. Hence, we designed a novel MLP to directly support the medial column. The MLP is contoured to the anatomy of the medial aspect of the proximal humerus and fixed by locking screws.

The construct stiffness is one measure of the integral stability of the whole subject. The current results showed that the LPMP significantly enhanced integral stability compared with the LP. Moreover, the LPMP provided stronger stability than the LPSG and LPDP methods, except for shear loads under Nor conditions. This can be attributed to the medial and lateral mechanical pathway and multi-planar fixation provided by the LPMP (Fig. 10). The fracture regional stability was assessed by evaluating the amplitude of distance and angle of the fracture gap. The LPMP provided the greatest antirotational stability under both bone conditions and provided anticompression/shear stability under Nor conditions due to direct dual-column support and center symmetrical fixation. The LPMP, LPSG, and LPDP methods all showed similar anticompression/shear ability under Ost conditions; this may be due to the weak holding force between the screws and the osteoporotic bone. In general, the LPMP provided strong construct stability. Strong fixation using the LPMP is beneficial for fracture healing, and patients can perform postoperative exercise earlier to recover shoulder joint function, except for motions that induce shear load.

The LPMP method resulted in the smallest change in NSA in all conditions. The changes in NSA under axial and shear loads were only respectively 0.5° and 3.9° under Nor conditions and 0.7° and 5.0° under Ost conditions. In the LPSG and LPDP methods, there is still a risk of varus malunion due to indirect medial support and fixation. Moreover, it is easy for varus malunion to develop when the LP and LPDP methods are used because of the unbalanced mechanical distribution caused by non-center symmetrical fixation. In addition to the fixation method, bone quality is also an important factor in varus collapse. Our results showed that the NSA of osteoporotic bone was less than that of normal bone under all conditions.

The maximum von Mises stresses for axial, shear, and torsional loads under Nor conditions on the LP were 505.0, 1181.1, and 906.5 MPa, respectively. The maximum value exceeded the material yield stress (800 MPa), which indicates a great risk of plastic yielding and fatigue cracking. The stresses were dispersed largely by additional implant/graft contact from locking plates when using the MLP, VA-LCP, and graft. The VA-LCP endured stresses of 1112.9 MPa, which also greatly exceeded the material yield stress. In contrast, the LPSG and LPMP were effective methods to prevent implant failure. Similar results occurred under Ost conditions; the stresses were markedly increased under shear loads. The disadvantage of increased shear load stresses is that patients cannot rise out of a chair or crutch weight-bear in the early postoperative period.

Calcar screws play an important role in providing medial support [12, 21]. The current study showed that the calcar screws endured heavy loads from the medial column. However, calcar screws were not used in the LPMP system. In real surgery, we performed minimally invasive surgery to place the lateral locking plate after euthyphoria medial column reduction and fixation by the medial approach and MLP. In addition, placing the calcar screws increases surgical trauma due to the cutting of the deltoid muscle. The current results showed that the LPMP provided strong integral and regional stability even without calcar screws. Park [22] reported a new technique that can place the lateral LP and MLP using only a single approach; this method could be used to place the calcar screws to increase the medial support effect.

There were limitations to the current study. First, as there is no standard biomechanical test for proximal humeral fracture [18], we used a biomechanical test method based on that used in previous research. Second, the finite element models were created based on the skeletal system, without considering the effect of muscles and ligaments, similarly to other finite element studies. Third, three- or four-part fractures were not created, as the experiment focus was on the medial buttress. Therefore, a 10-mm medial bone defect was used to simulate an unstable medial column. Fourth, only a single humeral model was used for analysis, which may avoid variation of interspecimen geometry and material characteristics. Fifth, although the shape and thickness of osteoporotic bone are very different to those of normal bone, we created the osteoporotic model only by changing the elastic modulus of the tissue, not by adjusting the shape or thickness. This is similar to the method used in previous research [23–25], and this method can still provide much information related to osteoporotic bone. Sixth, this study only evaluated early postoperative stability, without determining long-term biomechanical stabilization. Considering these limitations, the conclusions should be carefully used in clinical practice. Finally, we have to admit that this new generation of plate will cause extensive controversy. Our previous studies have proven that this method is clinically applicable. However, compared with other methods, the biomechanical property is not clear. Clinical characteristics should be considered deeply in further clinical studies. In the current paper, we only discuss biomechanical properties from the view of finite element analysis.

## Conclusions

In conclusion, this study provided evidence for some biomechanical advantages of this novel medial support technique. The LPMP provided high integral and regional construct stability under axial, shear, and torsional loads. The stresses on the lateral locking plate were dispersed largely by the MLP to prevent implant failure. The LPMP fixation provided strong medial buttressing, which may reduce the incidence of varus malunion and screw perforation. In general, finite element analysis showed that the LPMP was an effective treatment of proximal humeral fracture with an unstable medial column.

## Abbreviations

LP: Locking plate alone
LPDP: Locking plate combined with a distal radius plate
LPMP: Locking plate combined with medial anatomical locking plate
LPSG: Locking plate combined with a fibular graft
MLP: Medial anatomical locking plate
Nor: Normal bone condition
NSA: Neck-shaft angle
Ost: Osteoporotic bone condition
VA-LCP: Variable angle locking compression plate distal radius system
